# Macrosomie fœtale à Lubumbashi: facteurs de risque et pronostic maternel et périnatal

**DOI:** 10.11604/pamj.2016.23.166.7362

**Published:** 2016-04-06

**Authors:** Prosper Kakudji Luhete, Olivier Mukuku, Patrick Mubinda Kiopin, Albert Mwembo Tambwe, Prosper Kalenga Muenze Kayamba

**Affiliations:** 1 Département de Gynécologie-Obstétrique, Faculté de Médecine, Université de Lubumbashi, République Démocratique du Congo

**Keywords:** Macrosomie fœtale, facteurs de risque, pronostic maternel périnatal, Fetal macrosomia, risk factors, maternal complications, perinatal complications, Lubumbashi

## Abstract

**Introduction:**

La macrosomie fœtale est habituellement définie par un poids de naissance supérieur ou égal à 4000 grammes. L'objectif de cette étude est de déterminer la fréquence de la macrosomie, d'identifier les facteurs étiologiques et d’évaluer le pronostic maternel et périnatal.

**Méthodes:**

Il s'agit d'une étude cas-témoins menéeau sein des maternités de 10 hôpitaux généraux de référence de la ville de Lubumbashi en République Démocratique du Congoentre le 1er décembre 2013 et le 31 mars 2014. Les accouchées ont été réparties en deux groupes, en fonction du poids de naissance de leurs nouveau-nés: groupe I (≥4000 grammes ou plus) et groupe II (2500 à 3500 grammes). Les caractéristiques maternelles, l'environnement obstétrical ainsi que le pronostic maternel et périnatal ont été étudiés et comparés dans les deux groupes. Les données ont été analysées à l'aide du logiciel Épi info version 7.1. Les différences étaient jugées significatives pour un seuil de p<0,05.

**Résultats:**

Au total, 668 mères et leurs nouveau-nés ont été inclus dont 167 macrosomes et 501témoins. L'incidence de la macrosomie fœtale était de 5,7%. Comparativement aux mères de témoins, nous avons trouvé que les mères des nouveau-nés macrosomes étaient plus âgées, multipares, multigestes, obèses, diabétiques et avaient antérieurement donné naissance à un macrosome. Les taux de césarienne et de délivrance pathologique étaient significativement élevés chez les mères de macrosomes que chez celles de témoins. Lesexe masculin était significativement plus prédominant chez macrosomes que chez les témoins. La dystocie des épaules étaitenregistrée uniquement dans le groupe des macrosomes.

**Conclusion:**

La prévalence de l'accouchement d'un macrosome à Lubumbashi est de 5,7%. La macrosomie est souvent à l'origine de complications maternelles et périnatales. La réduction de ces dernières passe par une meilleure connaissance des facteurs de risque et un dépistage précoce.

## Abstract (en)

**Introduction:**

Fetal macrosomia is usually defined when the estimated fetal weight is greater than or equal to 4000 grams. The aim of this study was to determine the incidence of macrosomia, to identify its etiological factors and to evaluate maternal and perinatal prognosis.

**Methods:**

This is a case-control study conducted in maternity units of 10 general referral hospitals in the city of Lubumbashi in Democratic Republic of Congo between 1 December 2013 and 31 March 2014. The post-partum women were divided into two groups according to their fetal birth weight: group I (≥4000 grams or more) and groups II (from 2500 to 3500 grams). Maternal characteristics, obstetrical environment as well as maternal and perinatal prognosis were studied and compared in the two groups. Data were analyzed using Epi Info 7.1 software. Differences were considered significant if p <0,05.

**Results:**

A total of 668 mothers-newborns pairs were included in the study, 167 macrosoma and 501 controls. The incidence of fetal macrosomia was 5,7%. Compared to mothers from the control group, we found that mothers of macrosomic infants were older, multiparous, multigravidae, obese, diabetic and had previously delivered a macrosomic fetus. The rates of cesarean delivery and pathological delivery were significantly higher in mothers of macrosomic infants than in those of the control group. Male sex was significantly more prevalent among macrosomic infants than among the control group. Shoulder dystocia was recorded only in the macrosomic group.

**Conclusion:**

The prevalence of delivery of a macrosomic infant in Lubumbashi is 5,7%. Macrosomia is often the cause of maternal and perinatal complications. Reduction of maternal and perinatal complications passes through a better understanding of risk factors and an early detection.

## Introduction

Tout nouveau-né dont le poids de naissance est supérieur au 90èmepercentile des courbes de référence pour son âge gestationnel est appelé macrosome [1]. Généralement définie par un poids de naissance supérieur à 4000 grammes [2, 3], la macrosomie représente entre 2,4% et 24% de l´ensemble des accouchements [4–6] et de ce fait, elle constitue, non seulement une préoccupation permanente dans la pratique quotidienne de l´obstétricien, mais aussi dans celle du néonatologiste.

L'accouchement d'un macrosome comporte des complications maternelles et néonatales qui sont bien connues: la dystocie des épaules avec dans des rares cas une élongation du plexus brachial, l'asphyxie lors de l'expulsion, les fractures (clavicules, humérus) lors des manœuvres ainsi que l'hypoglycémie et l'hypocalcémie sur le plan néonatal; l'augmentation des césariennes (avant et pendant le travail), les lésions de la filiaire génitale immédiates (déchirures) ou lointaines (fistules) lors des accouchements par voie basse, les hémorragies du post-partum et les infections postpartales sont à noter sur le plan maternel [3, 5]. Les facteurs étiologiques de macrosomie fœtale sont nombreux et souvent intriqués et leur influence relative reste mal connue [7]. Nous ne possédons pas de données récentes sur le sujet en République Démocratique du Congo (RDC). A travers cette étude qui est la première à être consacrée à la macrosomie à Lubumbashi en RDC, nous nous sommes fixés comme objectifs de déterminer la fréquence de la macrosomie, d'identifier les facteurs étiologiques et d’évaluer le pronostic maternel et périnatal.

## Méthodes

Il s'agit d'une étude cas-témoin, réalisée dans la ville de Lubumbashi dans 10 maternités de référence sur une période de six mois du 1er décembre 2013 au 31 mai 2014. Au cours de cette période d’étude, nous avons enregistré tous les accouchements réalisés dans les maternités de 10 hôpitaux généraux de référence (HGR) de la ville de Lubumbashi en RD Congo (hôpital militaire de Ruashi, Cliniques Universitaires, hôpital Jason Sendwe, HGR Katuba, HGR Kenya, HGR Kamalondo, HGR Kisanga, HGR Kampemba, hôpital Gécamines-Sud et hôpital SNCC). Ces hôpitaux sont répartis dans les 7 communes que compte la ville de Lubumbashi (en RDC).

Cette étude a porté sur 167 accouchements des macrosomes (poids de naissance ≤4000 grammes) enregistrés de manière consécutive au cours de la période d’étude. Elle a comparé leur pronostic maternel et périnatal à celui des accouchements à terme de nouveau-nés dont le poids de naissance était compris entre 2500 et 3500 grammes (témoins). Le recrutement de témoins était fait par tirage de trois accouchements suivant l'accouchement d'un macrosome. Ainsi ce groupe a compté 501 témoins. N'ont pas été inclus dans cette étude, les accouchements survenus en dehors des maternités concernées par l’étude, les accouchements prématurés, les grossesses multiples et les fœtus porteurs de malformations.

Nous avons analysé pour la mère, les caractéristiques sociodémographiques, les antécédents médicaux et obstétricaux, la pression artérielle prise à l'accouchement, le mode d'accouchement, les complications et les suites de couches. L'obésité a été définie par l'indice de masse corporelle (IMC) supérieur ou égal à 30 kg/m^2^ et le surpoids par un IMC compris 25 et 29,9 kg/m^2^. L'hypertension artérielle (HTA) a été définie par une pression artérielle supérieure ou égale à 140/90 mmHg, mesure prise à deux fois à intervalle de 15 minutes, la femme étant au repos. La délivrance pathologique était définie par une délivrance marquée par une rétention placentaire, une atonie utérine ou une hémorragie de la délivrance. Pour le nouveau-né, nous avons analysé le score d'Apgar, le poids, le sexe, les complications néonatales et l'issue périnatale.

Les données ont été collectées sur une fiche d'enquête et recueillies par le personnel effectuant habituellement l´accouchement dans les sites d´enquête. Les données recueillies ont été analysées sur le logiciel Epi Info version 7.1. La comparaison entre les groupes des variables quantitatives a été faite en utilisant le test t de Student et le test de Mantel-Haenszela servi à comparer les variables qualitatives. Les ajustements ont été réalisés par la méthode de régression logistique pour les facteurs atteignant un degré de signification de p<0,2. Le seuil de signification a été fixé à p<0,05.

## Résultats

### Fréquence

**Figure 1:**
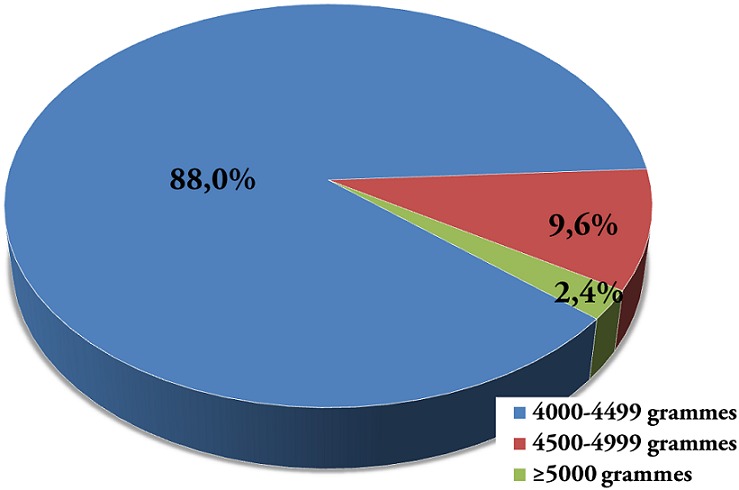
Distribution de macrosomes selon le poids de naissance

Sur un total de 2911 accouchées enregistrées au cours de la période d’étude, 167 avaient donné naissance à un macrosome soit une fréquence de 5,7%. De ces 167 macrosomes, 2,4% avaient un poids ≥5000 grammes et 9,6% pesaient entre 4500-4999 grammes (Figure 1). La moyenne de poids de naissance de macrosomes était de 4207,3±297,8 grammes (extrêmes: 4000 et 6400 grammes). Figure 1: distribution de macrosomes selon le poids de naissance

### Caractéristiques maternelles (Tableau 1)

La moyenne de l’âge maternel est de 30,0±6,0 ans (extrêmes: 16 et 46 ans) chez les mères des macrosomes alors qu'elle est de 28,3±6,3 ans (extrêmes: 15 et 46 ans) chez les mères des témoins. Le test t de Student montre une différence significative entre ces deux moyennes (p=0,0025). Le Tableau 1 montre que les proportions des accouchées âgées de 30 ans ou plus étaient de 52,1% chez les mères de macrosomes contre 41,9% chez les mères témoins. La comparaison de ces proportions donne une différence statistiquement significative (p=0,0219) signifiant que l’âge est significativement associé à l'accouchement d'un macrosome avec un risque de 1,5 fois pour un âge ≥30 ans (OR ajusté=1,5; IC95%:1,1-2,1). La parité et la gestité varient respectivement de 1 à 13 et de 1 à 15 dans les deux groupes autour des moyennes respectives de 4,7±2,5 et 5,1±2,8 chez les mères de macrosomes et de 3,8±2,4 et 4,1±2,7 chez les mères de témoins (p<0,0001). Il ressort du Tableau 1 que les proportions de multipares et des multigestes étaient plus élevées chez les mères de macrosomes que chez les mères de témoins (89,2% et 90,4% versus 79,4 et 83,5%; p<0,005) et la comparaison de ces proportions montre que les multipares et les multigestes présentent respectivement des risques de 2,1 et 2,2 fois de donner naissance à des macrosomes comparativement aux primipares et aux primigestes.

**Tableau 1:** Répartition des accouchées selon les caractéristiques sociodémographiques, les antécédents médicaux et obstétricaux

Variable	≥4000 g (n=167)		2500-3499 g (n=501)		p	ORajusté [IC95%]
	n	%	n	%		
Age maternel ≥30 ans	87	52,1	210	41,9	0,0223	1,5 [1,1-2,1]
Parité≥2	149	89,2	398	79,4	0,0052	2,1 [1,3-3,7]
Gestité ≥2	151	90,4	406	81,0	0,0048	2,2 [1,3-3,9]
Etat-civil« marié »	164	98,2	487	97,2	0,4785	1,6 [0,4-5,5]
Macrosomie antérieure	16	9,6	5	1,0	<0,0001	10,5 [3,8-29,2]
Césarienne antérieure	9	5,4	11	2,2	0,0423	2,5 [1,0-6,2]
Diabète sucré préexistant	2	1,2	0	0,0	0,0142	-
HTA préexistante	1	0,6	2	0,4	1,000	0,6 [0,05-7,4]
Obésité*	23	17,7	25	5,4	<0,0001	3,6 [2,0-6,6]

* L'indice de masse corporelle était précisé chez 130 accouchées du groupe I et chez 460 accouchées du groupe II.

En ce qui concerne les antécédents médicaux et obstétricaux, comparativement aux mères de témoins, les mères de macrosomes ont plus significativement un accouchement antérieur d'un macrosome, un accouchement antérieur par césarienne, un diabète sucré préexistant et une obésité (p<0,05). L'IMC moyen est de 26,5±3,7 kg/m^2^(extrêmes: 19,5 et 39,5 kg/m^2^) chez les mères de macrosomes contre 25,3±2,7 kg/m^2^ (extrêmes: 16,7 et 35,9 kg/m^2^) chez les mères de témoins; lorsque l'on compare ces deux moyennes, le test de Student montre que l'IMC moyen de mères de macrosomes est significativement plus élevé que celui de mères de témoins (p<0,0001). En revanche l’état-civil et l'antécédent d'HTA n'avaient pas d'influence sur la naissance d'un macrosome (p>0,05).

### Environnement obstétrical et complications maternelles (Tableau 2)

L'accouchement de macrosomes était fait par césarienne dans 15,6% contre 9,8% pour celui des témoins; nous avons noté un risque de près de 2 fois chez les macrosomes (OR ajusté=1,7; IC95%: 1,0-2,8; p=0,0419). La délivrance était plus significativement pathologique chez les mères de macrosomes que chez les mères de témoins (3,0% versus 0,4%; OR ajusté=7,7; IC95%:1,5-40,1; p=0,0153). La dystocie des épaules était observée à une proportion de 2,4% (4 cas)dans le groupe de macrosomes contre aucun cas dans le groupe témoin et l'analyse montre une différence statistique significative (p=0,0005).

**Tableau 2:** Distribution des accouchées selon l'environnement obstétrical et les complications maternelles

Variable	≥4000 g (n=167)		2500-3499 g (n=501)		p	OR ajusté [IC95%]
	n	%	n	%		
Présentation fœtale vicieuse	8	4,8	17	3,4	0,5562	1,4 [0,6-3,4]
Rupture prématurée des membranes	26	15,6	112	22,4	0,0621	0,6 [0,4-1,0]
Accouchement par césarienne	26	15,6	49	9,8	0,0419	1,7 [1,0-2,8]
Lésions des parties molles	14	8,4	35	7,0	0,6683	1,2 [0,6-2,3]
Episiotomie	11	6,6	49	9,8	0,2112	0,7 [0,3-1,2]
Délivrance pathologique	5	3,0	2	0,4	0,0153	7,7 [1,5-40,1]
Utilisation intrapartale d'ocytocine	51	30,5	129	25,7	0,2269	1,3 [0,9-1,9]
Dystocie des épaules	4	2,4	0	0,0	0,0005	indéfini
Rupture utérine	1	0,6	3	0,8	1,0000	0,7 [0,08-6,7]
Transfusion per ou post-partale	4	2,4	9	1,8	0,6275	1,3 [0,4-4,4]
Anémie post-partale	4	2,4	4	0,8	0,1006	3,0 [0,8-12,3]
Hémorragie post-partale	3	1,8	2	0,4	0,0979	4,6 [0,8-27,6]
Infection post-partale	1	0,6	2	0,4	0,7385	1,5 [0,1-16,7]

Dans le groupe des mères de macrosomes, cliniquement, nous avons enregistré des proportions élevées de présentation fœtale vicieuse, de lésions de parties molles, d'utilisation intrapartale d'ocytocine, de transfusion per ou post-partale, d'anémie, d'hémorragie et d'infection en post-partum mais l'analyse statistique n'a pas trouvé de différence significative comparativement au groupe témoin (p>0,05).

### Sexe des nouveau-nés et complications périnatales (Tableau 3)

Les proportions de sexe masculin étaient de 61,7% et 48,9% respectivement chez les macrosomes et les témoins; la comparaison de ces deux proportions montre une différence statistiquement significative en faveur d'une prédominance masculine chez les macrosomes (OR ajusté=1,7; IC95%: 1,2-2,4; p=0,0044). S'agissant des complications périnatales, bien que le groupe de macrosomes aient enregistré cliniquement des proportions élevées de mort fœtale in utéro, de dépression néonatale (score d'Apgar à la 5ème minute <7), de lésions traumatiques et de circulaire du cordon, l'analyse ne montre pas de différence statistiquement significative en comparaison du groupe témoin (p>0,05).

**Tableau 3:** Répartition des groupes selon le sexe des nouveau-nés et les complications périnatales

Variable	≥4000 g (n=167)		2500-3499 g (n=501)		p	OR ajusté [IC95%]
	n	%	n	%		
Sexe masculin	103	61,7	245	48,9	0,0044	1,7 [1,2-2,4]
Circulaire du cordon	3	1,8	1	0,2	0,0559	9,1 [0,9-88,5]
Score d'Apgar à la 5^ème^ minute <7	17	10,6	35	7,1	0,1637	1,5 [0,8-2,8]
Lésions traumatiques	1	0,6	2	0,4	0,5729	1,5 [0,1-16,9]
Décès périnatal	7	4,2	15	3,0	0,4526	1,4 [0,6-3,5]
Mort fœtale in utéro	6	3,4	9	1,9	0,1751	2,0 [0,7-5,8]
Décès intrapartal	1	0,6	3	0,6	1,000	1,0 [0,1-9,7]
Décès néonatal précoce	0	0,0	3	0,6	0,3165	indéfini

## Discussion

La présente étude relève une fréquence de 5,7% des accouchements des macrosomes. Cette fréquence se rapproche de 5,6%, 6,6% et 7% rapportées respectivement par Saleh en Arabie Saudite [8], par Fuchs en France [9] et par Akin en Turquie [10]. Elle est supérieure à celles rapportées dans certaines études africaines qui ont trouvé 1,57%, 2,1% et 2,4% respectivement au Sénégal, au Burkina-Faso et à Kinshasa (RDC) [4, 11, 12]. Des fréquences plus élevées supérieures à 20% ont été rapportées entre 1985 et 1998 au Canada (24%) [6] et entre 1992 et 1996 au Danemark (28%) [13]. Dans une revue de littérature américaine sur la macrosomie, Chauhan trouve que cette fréquence variait autour de 10% entre 1996 et 2002 [14]. Une étude chinoise enregistre une fréquence de 3,4% [15]. L´incidence de la macrosomie est différemment rapportée selon les différences raciales, les différences ethniques et la présence de facteurs locaux dans différentes régions [16]. Selon Cheng, la différence de distribution de poids à la naissance est probablement due à des différences génétiques et des anomalies anthropométriques entre les populations [5]. Des études épidémiologiques ont montré que les nourrissons chinois et sud-asiatiques sont plus petits pour leur âge gestationnel [17]. Les taux bas dans les études africaines pourraient s'expliquer par leur caractère monocentrique. Mais en plus, les facteurs de sous nutrition, de suivi insuffisant, de manque d'hygiène au cours de la grossesse, de bas niveau socio-économique peuvent expliquer ces taux bas.

L’âge maternel ≥30 ans est significativement associé à la naissance d'un macrosome dans notre série. Le poids de naissance augmente proportionnellement avec l’âge maternel. D'autres études trouvent que les mères des macrosomes sont plus âgées que celles des nouveau-nés normopondérés [10, 18–20]. Ce serait l'expression discrète de l'obésité ou du diabète dont le risque augmente avec l’âge. La multiparité, étant un facteur acquis expose à une augmentation du risque de macrosomie d'autant plus qu'il est souvent associé à l’âge. La plupart des travaux sont d'accord avec cette prédominance des multipares [18, 21, 22].

Notre étude a montré que le diabète et l'obésité constituaient des facteurs de risque de la macrosomie. Ce constat rejoint celui fait par plusieurs auteurs [10, 18, 23, 24]. Ceci s'explique par le mécanisme d'interdépendance du métabolisme des glucides et des lipides qui serait responsable d'un hyperinsulinisme fœtal réactionnel à l'hyperglycémie maternelle. L'insuline, hormone anabolisante, fait pénétrer les glucides dans les cellules, accumule les acides gras au niveau du tissu adipeux et les protéines dans les muscles entrainant ainsi la macrosomie fœtale [7, 25].

Comme dans d'autres études [18, 20, 22], notre étude avait montré que l'antécédent de macrosomie était également un facteurde risque de la macrosomie. Cette notion reste la plus constante de tous les facteurs de risque.

Il y avait significativement une prédominance masculine (61%) dans notre groupe d´étude. La prédominance du sexe masculin est indiscutable et plusieurs travaux rapportent des taux au-delà de 60% des cas [4, 18, 26]. Tous les auteurs s'accordent sur le fait que les nouveau-nés de sexe masculin pèsent généralement plus de nouveau-nés de sexe féminin et ceci à tout âge gestationnel.

Nos résultats avaient montré un taux significativement plus élevé de césarienne chez les mères de macrosomes que chez les mères des témoins (15,6 contre 9,8%). Ce taux de césarienne dans le groupe de macrosome est identique à celui trouvé par Fuchs [9]. Les données de la littérature montrent que la macrosomie est associée à un risque élevé de césarienne et des taux plus élevés au nôtre ont été rapportés dans la littérature allant de 21,4% à 51,4% [22, 27, 28]. Cette différence entre les taux de césarienne s'expliquerait par le fait que la macrosomie ne fait appel à la césarienne qu´après échec de l´épreuve du travail dans notre milieu alors qu´elle est directement sanctionnée par la césarienne en cas de présentation du siège. Selon Magnin, l'indication de la césarienne prophylactique pour prévenir une dystocie des épaules ne se discute pas dans les situations extrêmes comme un poids fœtal estimé supérieur à 5000 g ou supérieur à 4250 g chez une diabétique insulino-dépendante [29]. La dystocie des épaules était survenue dans 2,4% des cas dans le groupe des macrosomes contre un taux nul dans le groupe de comparaison dans notre série. Ce taux de dystocie des épaules chez les macrosomes est superposable à celui trouvé par El Barnoussi [30]. Batallan avait observé dix fois plus de dystocie des épaules chez les mères des macrosomes que chez les non macrosomes [31]. Il s'agit de la complication majeure la plus redoutable lors de l'accouchement d'un macrosome.

Bien que cliniquement nous ayons observé plus de morbidité néonatale dans le groupe de macrosomes que dans celui de non macrosomes, statistiquement nous n'avons pas noté de différence significative entre ces deux groupes. Les auteurs d'une enquête française multicentrique avaient conclu que la macrosomie ne s'accompagnait pas d'un excès de morbidité néonatale (traumatismes, score d'Apgar, pH au cordon, transfert néonatal) [31]. Les données de la littérature soutiennent que la morbidité maternelle et néonatale augmente avec le poids de naissance et surtout chez les nouveau-nés pesant plus de 4500 g [32–34].

## Conclusion

La prévalence de l'accouchement d'un macrosome à Lubumbashi est de 5,7%. La macrosomie est souvent à l'origine de complications maternelles et périnatales. La réduction de ces dernières passe par une meilleure connaissance des facteurs de risque et un dépistage précoce.

### Etat des connaissance sur le sujet

- La macrosomie fœtale constitue un problème majeur de santé publique en Afrique subsaharienne;
- Les complications maternelles périnatales restent élevées et les facteurs étiologiques sont nombreux et souvent intriqués.

### Contribution de notre étude à la connaissance

- Aucune étude sur ce sujet n'a déjà été publiée antérieurement sur les facteurs de risque et le pronostic maternel et périnatal de la macrosomie dans notre contexte, à Lubumbashi, République Démocratique du Congo;
- L’étude proposée est la première étude globale dans notre pays, intégrant une analyse multivariée permettant d’établir le caractère indépendant des facteurs de risque identifiés (ce qui est important pour en déduire des perspectives d'action);
- L’étude proposée est la première étude permettant d’évaluer le pronostic maternel et périnatal dans notre contexte.
